# Does previous asbestos exposure increase the risk of a post coronary artery bypass graft (CABG) pleural effusion – a routine data study?

**DOI:** 10.1186/s12890-023-02555-9

**Published:** 2023-08-21

**Authors:** Hugh Welch, Jessica Harris, Maria Pufulete, Arnaldo Dimagli, Umberto Benedetto, Nick Maskell

**Affiliations:** 1 University of Bristol, Bristol, UK; 2 Academic Respiratory Unit, North Bristol NHS Trust 2nd Floor Office, Learning and Research Building Southmead Hospital Southmead Way, Bristol, BS10 5NB UK

**Keywords:** Pleura, Pleural effusion, Pleural plaque, Asbestos, Coronary artery bypass graft, Routine data

## Abstract

**Background:**

Development of pleural effusion (PE) following CABG is common. Post-CABG PE are divided into early- (within 30 days of surgery) and delayed-onset (30 days–1 year) which are likely due to distinct pathological processes. Some experts suggest asbestos exposure may confer an independent risk for late-onset post-CABG PE, however no large studies have explored this potential association.

**Research question:**

To explore possible association between asbestos exposure and post-CABG PE using routine data.

**Methods:**

All patients who underwent CABG 01/04/2013–31/03/2018 were identified from the Hospital Episode Statistics (HES) Database. This England-wide population was evaluated for evidence of asbestos exposure, pleural plaques or asbestosis and a diagnosis of PE or PE-related procedure from 30 days to 1 year post-CABG. Patients with evidence of PE three months prior to CABG were excluded, as were patients with a new mesothelioma diagnosis.

**Results:**

68,150 patients were identified, of whom 1,003 (1%) were asbestos exposed and 2,377 (3%) developed late-onset PE. After adjusting for demographic data, Index of Multiple Deprivation and Charlson Co-morbidity Index, asbestos exposed patients had increased odds of PE diagnosis or related procedure such as thoracentesis or drainage (OR 1.35, 95% CI 1.03–1.76, p = 0.04). In those with evidence of PE requiring procedure alone, the adjusted OR was 1.66 (95% CI 1.14–2.40, p = 0.01). Additional subgroup analysis of the 518 patients coded for pleural plaques and asbestosis alone revealed an adjusted OR of post-CABG PE requiring a procedure of 2.16 (95% CI 1.38–3.37, p = 0.002).

**Interpretation:**

This large-scale study demonstrates prior asbestos exposure is associated with modestly increased risk of post-CABG PE development. The risk association appears higher in patients with assigned clinical codes indicative of radiological evidence of asbestos exposure (pleural plaques or asbestosis). This association may fit with a possible inflammatory co-pathogenesis, with asbestos exposure ‘priming’ the pleura resulting in greater propensity for PE evolution following the physiological insult of CABG surgery. Further work, including prospective studies and clinicopathological correlation are suggested to explore this further.

**Supplementary Information:**

The online version contains supplementary material available at 10.1186/s12890-023-02555-9.

## Background

It is well recognised that patients may develop pleural effusions following coronary artery bypass graft (CABG) surgery [1–3]. Between 65 and 89% of CABG procedures result in the development of a pleural effusion, however most resolve spontaneously or with medical management [4}. The underlying aetiology of these effusions may be independent of the surgery or related to it. Pleural effusions occurring after CABG are divided into early (< 30 days post-surgery) or late (> 30 days post-surgery) [4, 5]. Late effusions can be subdivided into those with a clear medical cause, such as cardiac failure or infection, and those without, and are much less studied than early effusions [2]. The pleural fluid in late occurring effusions, characterised in a 26 patient series, tends to be a lymphocytic exudate with normal glucose levels and lactate dehydrogenase (LDH) levels similar to the upper level of normal serum value [6].

The pathophysiology of the post-CABG pleural effusion is not well understood. It is postulated that the physiological insult of surgery may result in an inflammatory state [7]. This theory is supported by the finding of pro-inflammatory mediators such as interleukin-6 (Il-6) in pleural fluid in the post-operative phase [8]. Transforming growth factor-beta (TGF-beta) and vascular endothelial growth factor (VEGF) have been found to rise sequentially in pleural fluid in the perioperative period [9], suggesting an inflammatory cascade leading to altered pleural permeability, in turn resulting in pleural effusion formation. The influence and persistence in pleural fluid of VEGF is pertinent given its role in angiogenesis and vascular permeability [10]. Persistent post-CABG effusions have been observed to lead to diffuse pleural thickening (DPT), with corresponding histological evidence of chronic inflammation [11, 12].

Asbestos is known to cause lung and pleural injury and is associated with a variety of thoracic pathologies driven by inflammatory processes [13, 14]. Unpublished observational data from a large tertiary pleural centre suggests a possible correlation between asbestos exposure and the development of late onset post-CABG pleural effusion. The authors wished to explore this association further and ascertain if such a correlation could be observed in a national dataset. As both post-CABG pleural effusions and asbestos related lung disease appear to have inflammatory pathogenesis it is possible that previous asbestos exposure may confer an additional risk by ‘priming’ the pleural space prior to the additional inflammatory insult in the post CABG period. A similar effect has been postulated in the development of DPT following transudative effusions, and in bromocriptine-induced pleural effusions in asbestos-exposed individuals [15, 16], and DPT has been noted to be associated with increased rates of CABG surgery than in the baseline population [17].

## Methods

### Study design

In order to evaluate the relationship between asbestos exposure and late onset post-CABG pleural effusions, a retrospective population-based cohort study was undertaken. The data source used was the Health Episode Statistics (HES) for England database [18], a fully anonymised dataset which records all hospital admissions, outpatient appointments and Emergency Department attendances in NHS hospitals in England from 1997 onwards and has been validated as a research tool [19]. Data collected include patient demographic information, clinical information including diagnoses (using International Classification of Diseases (ICD-10) diagnosis codes) and procedures (Office of Population Censuses and Surveys (OPCS) procedure codes), administrative details of admissions and geographical data [20]. The University of Bristol holds a rolling ten-year licence for access to the dataset therefore the time period investigated was 1st April 2011 to 31st March 2019.

### Population

The population investigated was all adult patients who underwent CABG with or without concurrent heart valve repair or replacement. The OPCS procedure codes used to identify these patients are shown in Table 1. Relevant OPCS codes were selected with guidance from the Clinical Coding Department at Southmead Hospital, Bristol, and a cardiac surgeon. To maximise the likelihood of asbestos exposure being captured only patients with a minimum of three years of data in the dataset were included. As post-CABG effusions are thought to develop within one year of surgery, patients with less than one year of follow up data after their CABG were excluded. Patients with a record of pleural effusion diagnosis in the three months prior to CABG were also excluded.

### Exposure

Evidence of asbestos exposure was achieved by scrutinising target population HES records for recorded evidence of asbestos exposure in the three years prior to CABG procedure. The HES dataset does not reliably capture asbestos exposure as a discrete entity, thus a variety of diagnoses indicative of asbestos exposure were included in the search strategy. With the assistance of the Clinical Coding Department of Southmead Hospital and oversight from pleural physicians ICD-10 codes denoting asbestos exposure were identified (Table 1) [21]. English ICD-10 codes of pathologies associated with asbestos exposure were selected, the most prominent of which was the presence of calcified pleural plaques, which are pathognomonic of previous asbestos exposure. The search period of three years prior to CABG procedure maximised the likelihood of asbestos exposure being coded for on a patient’s record whilst ensuring that the number of patients included in the study was not excessively reduced.

### Outcomes

The outcome measured was the development of a pleural effusion between 30 days and 1 year post-CABG. To maximise data capture, the dataset was searched for both ICD-10 codes related to pleural effusions and OPCS codes related to medical procedures for pleural effusions. Two outcomes were assessed:

1. Presence of pleural effusion denoted by either primary or secondary CD-10 diagnosis or OPCS procedure related to pleural effusion.
2. Presence of pleural effusion denoted by OPCS procedure code alone.

Outcome 1 was felt likely to generate the largest number of results as the inclusion of both ICD-10 and OPCS codes allows for the capture of any patients in the target population who are found to have either a small pleural effusion that may not require or be safe for intervention, and those that required intervention. This outcome also allows for the capture of patients who may have undergone pleural intervention that has not been captured by the coding database. Patients with both ICD-10 codes for pleural effusion and OPCS codes for procedure were counted as single incidences of pleural effusion. However, the majority of late onset post-CABG effusions are sufficiently large to require intervention in the form of pleural aspiration or drainage [2]. Outcome 2 was chosen to provide a narrower search that would only identify larger effusions requiring intervention.

**Table 1:** OCPS and ICD-10 codes used to identify the population, exposure and evidence of pleural effusion in the HES Dataset

CABG OPCS Procedure Code:	
K40	Saphenous vein graft replacement of coronary artery
K41	Other autograft replacement of coronary artery
K42	Allograft replacement of coronary artery
K43	Prosthetic replacement of coronary artery
K44	Other replacement of coronary artery
K45	Connection of thoracic artery to coronary artery
K46	Other bypass of coronary artery
**ICD-10 diagnosis codes that indicate asbestos exposure**:	
J61	Pneumoconiosis due to asbestos and other mineral fibres
Z57.2	Occupational exposure to dust *
J92.0	Pleural plaque with presence of asbestos
J92.9	Pleural plaque without asbestos *
**OPCS Procedure codes that indicate pleural effusion**:	
T083	Fenestration of pleura
T121	Drainage of lesion of pleura NEC
T122	Drainage of pleural cavity NEC
T123	Aspiration of pleural cavity
T124	Insertion of tube drain into pleural cavity
T128	Other specified puncture of pleura
T129	Unspecified puncture of pleura
**ICD-10 diagnosis codes that indicate pleural effusion**:	
J90	Pleural effusion, not elsewhere classified
J91	Pleural effusion in conditions classified elsewhere

OPCS - Office of Population Census and Surveys; ICD-10 – International Classification of Disease version 10; HES – Health Episode Statistics

*= not included in all analyses

### Statistical methods

Summary statistics were presented as proportions and as medians and interquartile range (IQR) where data were skewed. Standardised mean differences (SMD) were calculated. A SMD of 0.10 was the threshold used to denote a meaningful imbalance in the covariates between groups [22]. Charlson comorbidity index (CCI) scores were calculated by reviewing all admissions in the one year prior to the CABG and using all episodes in the index admission up to the date of the CABG procedure. Appropriate scores were assigned according to the approach detailed elsewhere [23]. Each relevant diagnosis was counted only once for each patient; clinical conditions were scored and these scores were summed to provide a total score that predicts mortality. Logistic regression models were used to quantify the association between recorded asbestos exposure and pleural effusions and were adjusted for confounding factors decided *a priori*: age, sex, index of multiple deprivation (IMD), CCI and CABG procedure type. Patients with no hospital admissions in the three years prior to CABG were excluded from a subgroup analysis as no inpatient data was available on prior health conditions or asbestos exposure. All analyses were completed using Stata v17 (StataCorp LP, College Station, TX, USA).

## Results

### Characteristics

**Fig. 1:**
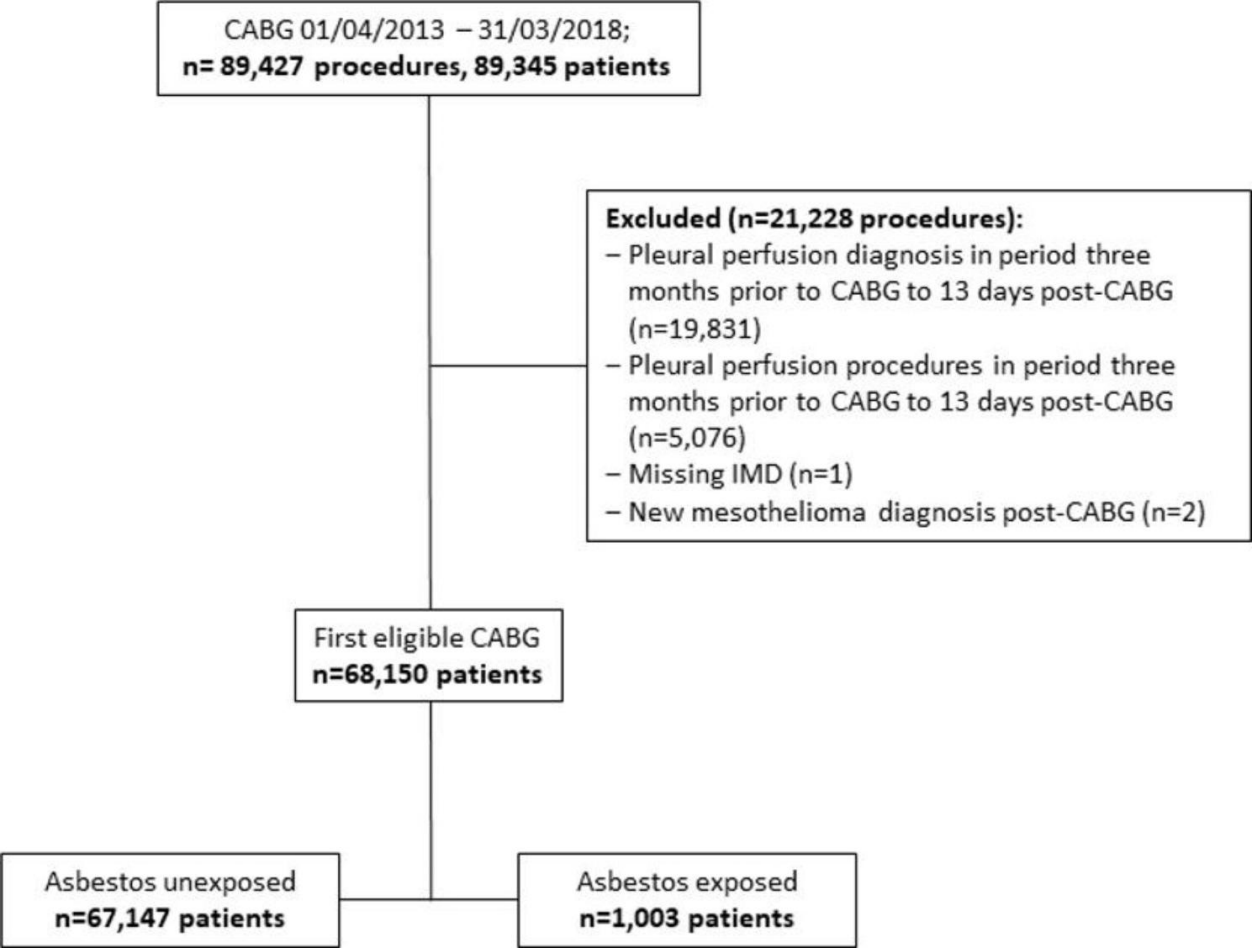
CONSORT diagram (CABG – Coronary artery bypass graft; IMD – index of multiple deprivation)

89,345 patients who had undergone 89,427 CABG procedures between 01/04/2018 and 31/03/2018 were identified. 19,514 procedures were excluded – 18,144 due to their records showing a pleural effusion diagnosis related code from 3 months prior to their CABG procedure. A further 4,006 records were excluded due to pleural effusion procedure codes in the same period. One record was excluded due to missing IMD data. Two cases of mesothelioma were identified and excluded. The remaining number of eligible records identified was 68,150 (See Fig. 1).

The mean age of the population was 68 years and the patients were predominantly male; 82% (n = 56,971) (Table 2). Patients were relatively evenly distributed across all IMD deciles, with between 8 and 11% in each decile. The median CCI was 1 (IQR 1–3).

Within this population, 1,035 (1.5%) patient records were found with ICD-10 codes suggestive of asbestos exposure. However, 16,874 patients in the unexposed cohort had had no hospital admissions in the three years prior to their CABG procedure so it is not possible to define asbestos exposure in this group.

**Table 2:** Patient demographics and CABG procedure details

Patient characteristics		Not exposed to asbestos (n = 67,147)	Exposed to asbestos (n = 1,003)	Total (n = 68,150)	SMD
Age; median (IQR)		68 (60, 75)	73 (69, 78)	68 (60, 75)	0.67
Sex; n (%)	Male	54,695 (81%)	973 (97%)	55,668 (82%)	0.52
	Female	12,452 (19%)	30 (3%)	12,482 (18%)	
IMD decile; n (%)	1 (most deprived)	6217 (9%)	108 (11%)	6325 (9%)	0.10
	2	6214 (9%)	91 (9%)	6305 (9%)	
	3	6365 (9%)	108 (11%)	6473 (9%)	
	4	6842 (10%)	106 (11%)	6948 (10%)	
	5	6925 (10%)	97 (10%)	7022 (10%)	
	6	7088 (11%)	101 (10%)	7189 (11%)	
	7	7092 (11%)	92 (9%)	7184 (11%)	
	8	6929 (10%)	102 (10%)	7031 (10%)	
	9	7009 (10%)	115 (11%)	7124 (10%)	
	10 (least deprived)	6466 (10%)	83 (8%)	6549 (10%)	
CCI; median (IQR)		1 (1, 3)	2 (1, 3)	1 (1, 3)	0.28
**CABG procedure**					
Year; n (%)	2013/14	14,884 (22%)	210 (21%)	15,094 (22%)	0.07
	2014/15	14,512 (22%)	211 (21%)	14,723 (22%)	
	2015/16	13,326 (20%)	190 (19%)	13,516 (20%)	
	2016/17	12,702 (19%)	192 (19%)	12,894 (19%)	
	2017/18	11,723 (17%)	200 (20%)	11,923 (17%)	
K40 Saphenous vein graft replacement of coronary artery; n (%)		59,113 (88%)	884 (88%)	59,997 (88%)	0.003
K41 Other autograft replacement of coronary artery; n (%)		3052 (5%)	24 (2%)	3076 (5%)	0.12
K42 Allograft replacement of coronary artery; n (%)		41 (0.1%)	-	41 (0.1%)	0.03
K43 Prosthetic replacement of coronary artery; n (%)		33 (0.1%)	1 (0%)	34 (0.1%)	0.02
K44 Other replacement of coronary artery; n (%)		374 (1%)	6 (1%)	380 (1%)	0.01
K45 Connection of thoracic artery to coronary artery; n (%)		59,306 (88%)	828 (83%)	60,134 (88%)	0.16
K46 Other bypass of coronary artery; n (%)		28 (0%)	-	28 (0%)	0.03

*CABG – Coronary Artery Bypass Graft; CCI – Charlson Co-morbidity Index; IMD – Index of Multiple Deprivation; IQR – interquartile range; SMD – standardised mean difference*

Demographic data were broadly similar across the two groups, apart from some notable differences (Table 2). Patients exposed to asbestos were on average five years older, with a larger proportion of men (97% vs. 81%) and more comorbidities (CCI 2 vs. 1). Although similar proportions of both groups underwent saphenous vein graft (SVG) insertion (88% in both groups), a lower proportion of the exposed group underwent thoracic artery, also known as internal mammary artery, connection to coronary artery (83% vs. 88%).

**Table 3:** Frequency of evidence of asbestos exposure and evidence of pleural effusion

Asbestos exposure and Pleural effusion		n = 68,150
Asbestos exposure diagnosis in 3 years before CABG; n (%)	Absent	67,147 (99%)
	Present	1003 (1%)
Pleural effusion diagnosis or procedure in 30 days to 1 year after CABG; n (%)	Absent	65,773 (97%)
	Present	2377 (3%)
Pleural effusion procedure in 30 days to 1 year after CABG; n (%)	Absent	67,188 (99%)
	Present	962 (1%)

CABG – coronary artery bypass graft

**Table 4:** Association between asbestos exposure and post-CABG pleural effusion

Outcome 1: Pleural effusion diagnosis or procedure in 30 days to 1 year after CABG	OR (95% CI)	p-value
Crude model	1.78 (1.37, 2.32)	< 0.001
Adjusted model (age, sex, IMD, CCI, CABG codes)1	1.35 (1.03, 1.76)	0.04
**Outcome 2: Pleural effusion procedure in 30 days to 1 year after CABG**	**OR (95% CI)**	**p-value**
Crude model	2.19 (1.51, 3.17)	< 0.001
Adjusted model (age, sex, IMD, CCI, CABG codes)2	1.66 (1.14, 2.40)	0.01

*CABG – Coronary Artery Bypass Graft; CCI – Charlson Co-morbidity Index; IMD – Index of Multiple Deprivation; OR – odds ratio; 95% CI – 95% confidence interval*

Pleural effusion diagnosis or related procedures recorded from 30 days to one year after CABG procedure were identified in 2,377 patients, with 962 cases of procedure alone, as shown in Table 3. The absolute numbers of procedures identified are presented in supplementary Table 1.

Both outcomes showed a modest increase in odds of pleural effusion development for patients with recorded asbestos exposure, in both crude and adjusted models (Table 4).

**Fig. 2:**
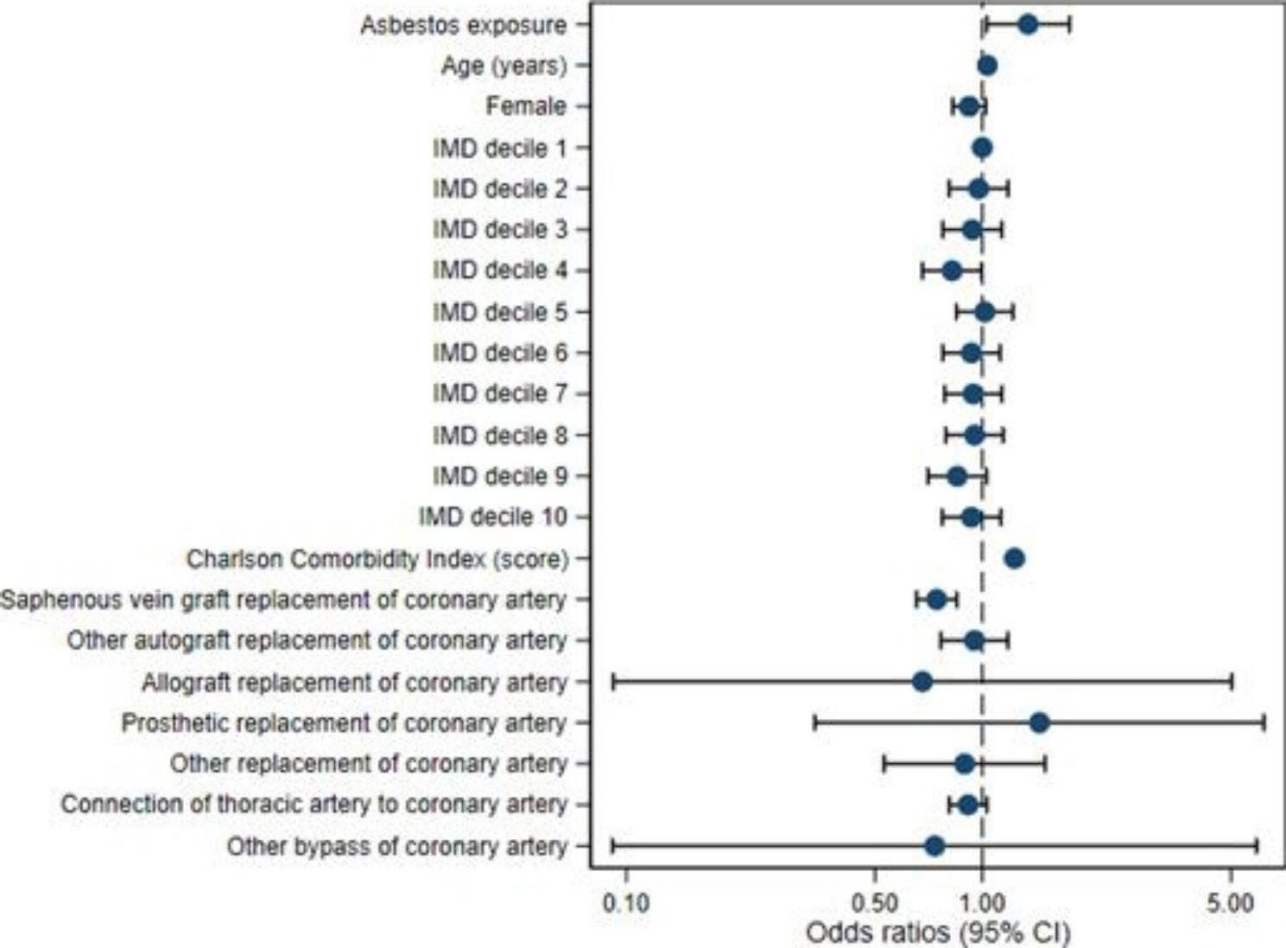
Adjusted model risk associations for Outcome 1: Pleural effusion diagnosis or procedure in 30 days to 1 year after CABG (IMD – Index of Multiple Deprivation; 95% CI – 95% confidence interval)

**Fig. 3:**
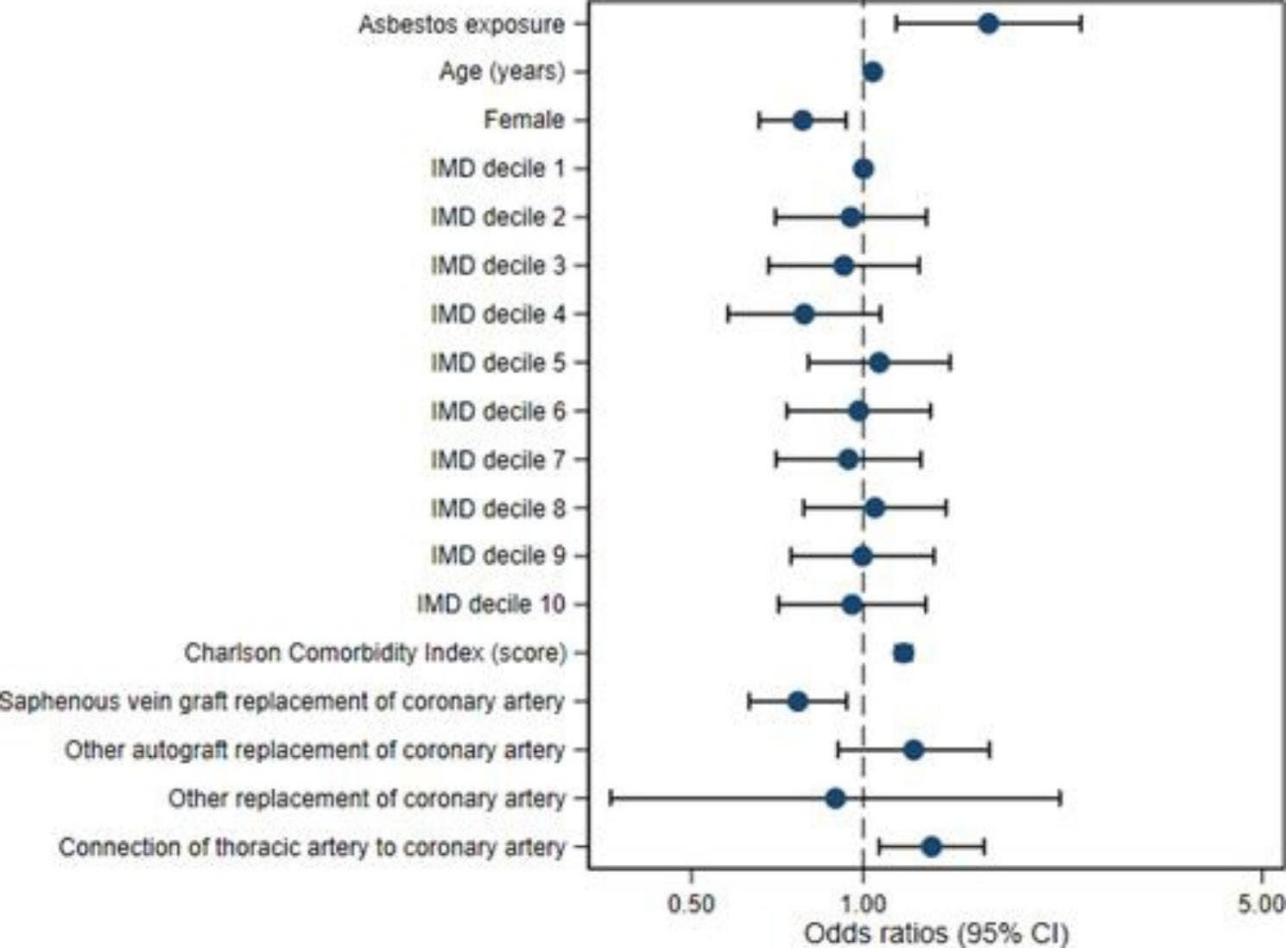
Adjusted model associations for Outcome 2: Pleural effusion procedure in 30 days to 1 year after CABG (IMD – Index of Multiple Deprivation; 95% CI – 95% confidence interval)

Figures 2 and 3 show adjusted model risk associations for both outcomes. Both demonstrate the increased odds ratio for pleural effusion denoted by asbestos exposure. Figure 3 shows increased odds ratios of pleural effusion development associated with asbestos, internal mammary artery usage and increasing CCI score. It also shows a decreased odds ratio denoted by female sex.

A sensitivity analysis was undertaken which excluded the 12,479 patients with no hospital admissions in the three years prior to CABG surgery. The results generated are similar to those in the main population and are shown in supplementary Table 2.

Although calcified pleural plaques are not known be caused by any other aetiology than asbestos exposure, the authors recognise that the use of the codes ICD-10 diagnostic codes Z57.2 (Occupational exposure to dust) and J92.9 (Pleural plaque without asbestos) may introduce a risk of erroneously identifying patients as asbestos exposed. Therefore, the analysis was repeated using only patients identified using the codes J61 (Pneumoconiosis due to asbestos and other mineral fibres) and J92.0 (Pleural plaque with presence of asbestos). It should be noted that other pneumoconioses secondary to dust exposure, such as silica and talc (J62) and other dusts such as aluminium, bauxite, beryllium or graphite (all J63) are listed under separate ICD10 codes. By using only J61 and J92.0, the number of asbestos exposed patients identified was reduced from 1003 to 518. The crude and adjusted results are shown in Table 5.

**Table 5:** Association between asbestos exposure and post-CABG effusion (J92.0 and J61 codes only)

Outcome 1: Pleural effusion diagnosis or procedure in 30 days to 1 year after CABG	OR (95% CI)	p-value
Crude model	2.27 (1.64, 3.16)	< 0.001
Adjusted model (age, sex, IMD, CCI, CABG codes)	1.63 (1.17, 2.28)	0.01
**Outcome 2: Pleural effusion procedure in 30 days to 1 year after CABG**	**OR (95% CI)**	
Crude model	2.99 (1.93, 4.66)	< 0.001
Adjusted model (age, sex, IMD, CCI, CABG codes)	2.16 (1.38, 3.37)	0.002

*CABG – Coronary Artery Bypass Graft; CCI – Charlson Co-morbidity Index; IMD – Index of Multiple Deprivation; OR – odds ratio; 95% CI – 95% confidence interval*

**Fig. 4:**
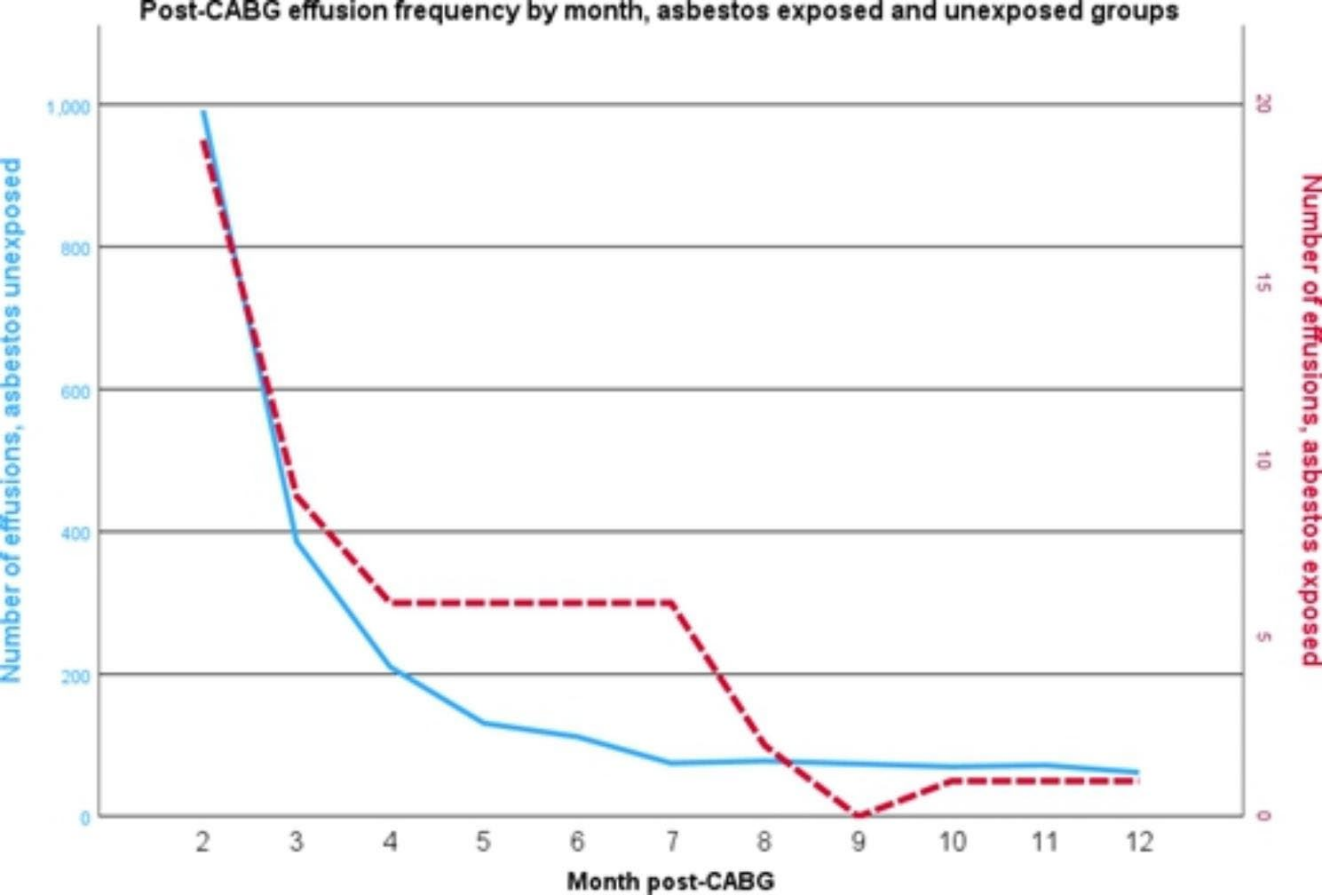
Post-CABG effusion frequency by month, asbestos exposed and unexposed groups. CABG – Coronary Artery Bypass Graft

The monthly frequency of pleural effusion development in the cohort is similar in both asbestos exposed and unexposed patients, with the majority of effusions occurring in the first 6 months post-operatively then tailing off over the remainder of the year (Fig. 4).

## Discussion

Although the use of asbestos was banned in the UK in 1999, many workers were exposed prior to this ban and more recently due to asbestos in the fabric of existing buildings being disturbed. This study explored the association between post-CABG pleural effusion and asbestos exposure. No other published work has addressed this question using such a large prospectively collected dataset.

The HES dataset allows for review of very large numbers of patient records. The population of 69,860 is an accurate number of patients who had undergone CABG surgery in the NHS in England for the time period 2013–2018, due to dataset design.

The demographic data of the study patients is in line with the age and sex of the general population. The higher incidence of asbestos exposure in males compared to females (n = 1003, 97%; n = 32, 3%) reflects the male predominance of asbestos-related disease [24]. Smoking status has not been evaluated in this study therefore it is not known whether the identified population reflects general population smoking trends. Type of CABG surgery undertaken across both groups is similar, although internal mammary artery connection is lower in the asbestos-exposed population, despite being the ‘gold standard’ approach were feasible [26, 27]. This reduces the potentially confounding influence of internal mammary artery usage, an approach associated with post CABG pleural effusion [25]. Internal mammary artery use is associated with a higher degree of morbidity and complication in females thus the approach is more frequently performed in males [25, 26].

Multiple authors have reported an association between late post-CABG pleural effusion and internal mammary artery graft usage [2, 27–29]. This is thought due to operative interruption of the pleura to access and utilise the vasculature. However, a retrospective study of 410 patients that found no difference in the rate of post-CABG pleural effusion in patients with internal mammary artery connections compared to those with saphenous vein grafts (SVG) [29].

Asbestos exposed patients are distributed throughout IMD deciles. Historically, occupations that lead to asbestos exposure were predominantly manual or ‘blue collar’, such as pipe laggers, shipyard workers and foundry workers. However, the even distribution of these patients suggests that individuals of all socioeconomic classes and occupations have been exposed to asbestos. This is in line with the observed shift in some countries towards institutional and ‘legacy’ asbestos exposure, with higher rates seen in professions such as teaching and elections [30, 31]. In contrast however, the strongly male (97%) preponderance for asbestos exposure is in line with established knowledge of asbestos exposure [32].

All outcomes from this study demonstrate modest increases in odds of development of a post-CABG pleural effusion in patients with documented asbestos exposure. Outcome 1, designed to capture as many patients as possible who have any record of a new pleural effusion diagnosis or procedure in the period of interest, revealed a crude odds ratio (OR) of 1.78 (95% CI 1.37–2.32; p < 0.001) and an adjusted OR of 1.35 (1.03–1.76; p = 0.04) for development of pleural effusion following asbestos exposure. This suggests that asbestos exposure is associated with a moderately higher risk of developing a post-CABG pleural effusion over those who are unexposed.

This finding is mirrored in Outcome 2, in which only procedures associated with pleural effusions are recorded. The crude OR demonstrated was 2.19 (1.51–3.17; p < 0.001) and adjusted OR was 1.66 (1.14–2.40; p = 0.01). This shows a stronger association between asbestos exposure and pleural effusion development, although the confidence intervals obtained are wider in this population. This is an expected finding and correlates well with supporting literature [27, 29].

The analysis focussing only on codes J61 and J92.0 produced the strongest evidence of risk association, with an adjusted OR of 2.16 (1.38–3.37; p = 0.002). In this group of patients, whose diagnostic codes reflect a sufficient degree of asbestos exposure to cause radiographic changes such as plaques or asbestosis, the odds of post-CABG effusion development is more than doubled compared to the control population.

The sensitivity analysis excluded 12,479 patients with no record of hospital admission in the three years prior to CABG. Due to the lack of hospital records it is impossible to define whether they have been exposed to asbestos or not. It is acknowledged that this approach may result in overestimation of the true rate of asbestos exposure. Outcome 1 in this group demonstrated a crude OR of 1.72 (1.32–2.24 p < 0.001) and adjusted OR of 1.35 (1.03–1.77 p = 0.03). For outcome 2 the crude OR was 2.12 (1.47–3.08 p < 0.001) and adjusted 1.65 (1.14–2.40 p = 0.01). These are very similar to the outcomes from the original analysis however the confidence intervals are wider, reflecting the smaller patient numbers and limiting the interpretation of these results. Nonetheless, they add weight to the association between asbestos exposure and post-CABG effusion. This sensitivity analysis excludes a large number of the ‘healthy’ population by excluding patients with no hospital attendances in the three years prior to CABG. Although this approach may generate a more representative incidence of asbestos exposure in the study population, it necessarily skews the population towards higher impact health service users with inherent higher degrees of illness and co-morbidity.

The frequency distribution of pleural effusion development over the 12 months post CABG is in keeping with the post-surgical effusion development hypothesis, rather than reflecting benign asbestos related effusion (BAPE) development. By excluding any mesothelioma diagnoses from the data we have significantly reduced the risks these pleural effusions have a malignant aetiology.

The proportion of procedures performed on patients in this study appears to fall in favour of chest drainage (n = 552) rather than pleural aspiration (n = 353). However, 209 patients are coded as having under gone ‘drainage of pleural cavity NEC’ and 26 ‘other specified puncture of pleura’. Whilst ‘drainage of pleural cavity NEC’ is likely to represent chest tube drainage rather than aspiration, this cannot be confirmed therefore it is not possible to interrogate these numbers further. Allowing for these 235 patients, the skew in favour of chest drainage likely represents both a larger number of symptomatic effusions and a potential reticence to perform aspirations on small effusions. Clinical experience suggests that small, asymptomatic effusions are not always be aspirated in the setting of CABG follow up - the treating clinician may feel the diagnosis is clear and no fluid analysis required.

This study has limitations that must be acknowledged when interpreting results. As a retrospective analysis, this work may only describe associations rather than causality. The relatively low numbers of patients identified and modest effect size should also be borne in mind by the reader when interpreting our findings.

Although the HES dataset allows for analysis of huge numbers of patient records, the way that the data is recorded and the manner in which clinical coding is undertaken in England result in inconsistent data capture for many medical procedures, especially those carried out during an inpatient admission – the details are often recorded in complex medical notes that may be missed by clinical coders. Elective surgical admissions, outpatient episodes and procedures result in better data capture due to the manner in which hospitals in England derive revenue streams. Subtle medical diagnoses and risk factors such as asbestos exposure may be incompletely and inconsistently recorded in medical notes and coding episodes. Therefore the HES dataset is likely to under-report the true number of patients with asbestos exposure and also may under-report the number of patients with pleural effusions requiring intervention. As the HES dataset is primarily a coding repository, clinical information such as test results are not recorded, therefore this study is unable to analyse clinical information including biochemical parameters of pleural fluid and imaging findings.

The HES dataset does not allow for the sizing of the effusions to be analysed, nor does it record the underlying aetiology. Thus it is not possible to guarantee that all reported effusions are post-CABG in origin. The exclusion of patients who had a diagnosis of pleural effusion in the 3 month prior to CABG reduces the probability of capturing an effusion due to another aetiology. Selection criteria for CABG surgery will also exclude patients with conditions such as malignant pleural effusion. Therefore, the authors believe that the vast majority of the pleural effusions reported in this study represent post CABG effusions, however this cannot be quantified. Allowing for this, the data reported here still show an increase in the incidence of pleural effusions of all causes in those with previous asbestos exposure.

### Interpretation

The results of this study show that there is a modest association between asbestos exposure and post-CABG pleural effusion. Asbestos exposure has been demonstrated to confer an adjusted OR of 1.66 for the development of a pleural effusion requiring intervention in the period 30 days − 1 year following CABG. This association is reflected in all analyses performed, albeit with variation in OR value and confidence intervals. In patients with radiological evidence of asbestos exposure (pleural plaques or asbestosis), the OR is 2.16 – representing a doubling of risk compared to unexposed patients. The study findings correlate with anecdotal experience from asbestos disease specialists and suggest a potential link between asbestos exposure and the development of last onset post-CABG pleural effusions. The possibility of shared inflammatory pathogenesis, with asbestos mediated ‘priming’ of the pleural space is raised. Finally, this study demonstrates the utility of the HES dataset in providing data for large scale study of relatively rare conditions that may not be adequately captured in local registries.

Further work using prospective collected detailed asbestos exposure data, chest radiograph analysis and pleural fluid analysis is now required. Additional clinicopathological studies are also needed to explore the aetiological links between the two entities.

### Electronic supplementary material

Below is the link to the electronic supplementary material.

Supplementary Material 1

## Data Availability

The datasets analysed during the current study are available under license in the NHS Digital repository https://digital.nhs.uk/data-and-information/data-tools-and-services/data-services/hospital-episode-statistics.

## Abbreviations

BAPE: Benign Asbestos-related Pleural Effusion
CABG: Coronary Artery Bypass Graft
CCI: Charlson Comorbidity Index
DPT: Diffuse Pleural Thickening
HES: Health Episode Statistics
ICD-10: International Classification of Diseases 10th Revision
IL-6: Interleukin 6
IMD: Index of Multiple Deprivation
OCPS: Office of Population Censuses and Surveys
OR: Odds Ratio
PE: Pleural Effusion
SMD: Standardised Mean Difference
TGF-beta: Transforming Growth Factor Beta
VEGF: Vascular Endothelial Growth Factor

## References

[1] Kollef MH, Peller T, Knodel MA (1988). Hal Cragun, *delayed Pleuropulmonary Complications following coronary artery revascularization with the Internal Mammary Artery*. Chest.

[2] Light RW (2002). Pleural effusions after coronary artery bypass graft surgery. Curr Opin Pulm Med.

[3] Hurlbut D, Lee Myers M, Lefcoe M, Goldbach M (1990). Pleuropulmonary morbidity: Internal thoracic artery versus saphenous vein. Ann Thorac Surg.

[4] Light RW, Rogers JT, Cheng D, Rodriguez RM (1999). Large pleural effusions occurring after coronary artery bypass grafting. Cardiovascular surgery associates, PC. Ann Intern Med.

[5] Heidecker J, Sahn SA (2006). The spectrum of pleural effusions after coronary artery bypass grafting surgery. Clin Chest Med.

[6] Sadikot RT, Rogers JT, Cheng D-s, Moyers P, Rodriguez M, Light RW (2000). f.t.C.S.A. PC, *Pleural Fluid characteristics of patients with symptomatic pleural effusion after coronary artery bypass graft surgery*. Arch Intern Med.

[7] Maisch B, Seferović PM, Ristić AD, Erbel R, Rienmüller R, Adler Y, Tomkowski WZ, Thiene G, Yacoub MH, Priori SG, Alonso Garcia MA, Blanc J-J, Budaj A, Cowie M, Dean V, Deckers J, Fernandez Burgos E, Lekakis J, Lindahl B, Mazzotta G, Moraies J, Oto A, Smiseth OA, Mazzotta G, Acar J, Arbustini E, Becker AE, Chiaranda G, Hasin Y, Jenni R, Klein W, Lang I, Lüscher TF, Pinto FJ, Shabetai R, Simoons ML, Soler Soler J, Spodick DH (2004). .f.P.G. Task Force members, Document Reviewers, *Guidelines on the diagnosis and management of Pericardial Diseases Executive Summary: the Task Force on the diagnosis and management of Pericardial Diseases of the European Society of Cardiology*. Eur Heart J.

[8] Chibante AM, Vaz MC, Vargas FS. *[IL-6 anti-inflammatory activity in pleural effusion post-coronary artery bypass graft surgery]* Rev Port Pneumol, 2007. 13(3): p. 319 – 34. 17632672

[9] Chibante AM, Vaz MA, Suso FV (2006). The proliferative cytokines TGF-beta and VEGF in pleural effusions post-coronary artery bypass graft. Rev Port Pneumol.

[10] Leung DW, Cachianes G, Kuang WJ, Goeddel DV, Ferrara N (1989). Vascular endothelial growth factor is a secreted angiogenic mitogen. Science.

[11] Lee YCG, McDonald EC, Nesbitt JC, Vaz MAC, Ely KA, Light RW, Thompson PJ (2001). Symptomatic persistent post-coronary artery bypass graft pleural Effusions requiring operative treatment: clinical and histologic features. Chest.

[12] Touma T, Taira R, Makida T, Oshiro K, Miyara T, Taba Y (2022). Marked ventilation impairment due to progression of diffuse pleural thickening after cardiac surgery. Radiol Case Rep.

[13] *A review of human carcinogens—Part C: metals, arsenic, dusts, and fibres* The Lancet Oncology, 2009. 10(5): p. 453–454. 10.1016/s1470-2045(09)70134-2 19418618

[14] Huggins JT, Sahn SA (2004). Causes and management of pleural fibrosis. Respirology.

[15] Evison M, Barber P (2015). Diffuse pleural thickening following heart failure-related pleural effusions in an asbestos exposed patient. Int J Occup Environ Health.

[16] Knoop C, Mairesse M, Lenclud C, Gevenois P, De Vuyst P (1997). Pleural effusion during bromocriptine exposure in two patients with pre-existing asbestos pleural plaques: a relationship?. Eur Respir J.

[17] Kee ST, Gamsu G, Blanc P (1996). Causes of pulmonary impairment in asbestos-exposed individuals with diffuse pleural thickening. Am J Respir Crit Care Med.

[18] [cited 2022 25/01/2022]. ; Available from: https://digital.nhs.uk/data-and-information/data-tools-and-services/data-services/hospital-episode-statistics.

[19] Thorn JC, Turner E, Hounsome L, Walsh E, Donovan JL, Verne J, Neal DE, Hamdy FC, Martin RM, Noble SM (2016). Validation of the Hospital Episode Statistics Outpatient dataset in England. PharmacoEconomics.

[20] World Health O. ICD-10: international statistical classification of diseases and related health problems : tenth revision. World Health Organization: Geneva; 2004.

[21] Bortolussi G, McNulty D, Waheed H, Mawhinney JA, Freemantle N, Pagano D (2019). Identifying cardiac surgery operations in hospital episode statistics administrative database, with an OPCS-based classification of procedures, validated against clinical data. BMJ Open.

[22] Mamdani M, Sykora K, Li P, Normand S-LT, Streiner DL, Austin PC, Rochon PA, Anderson GM (2005). Reader’s guide to critical appraisal of cohort studies: 2. Assessing potential for confounding. BMJ.

[23] Maringe C, Fowler H, Rachet B, Luque-Fernandez MA (2017). Reproducibility, reliability and validity of population-based administrative health data for the assessment of cancer non-related comorbidities. PLoS ONE.

[24] Light RW (2011). Pleural effusions. Med Clin North Am.

[25] Saraiva FA, Girerd N, Cerqueira RJ, Ferreira JP, Vilas-Boas N, Pinho P, Barros A, Amorim MJ, Lourenço AP (2018). Leite-Moreira, *Survival after bilateral internal mammary artery in coronary artery bypass grafting: are women at risk?*. Int J Cardiol.

[26] Koch CG, Nussmeier NA (2003). Gender and cardiac surgery. Anesthesiol Clin North Am.

[27] Labidi M, Baillot R, Dionne B, Lacasse Y, Maltais F, Boulet LP (2009). Pleural effusions following cardiac surgery: prevalence, risk factors, and clinical features. Chest.

[28] Charniot J-C, Zerhouni K, Kambouchner M, Martinod E, Vignat N, Azorin J, Gandjbakhch I, Artigou J-Y (2007). Persistent symptomatic pleural effusion following coronary bypass surgery: clinical and histologic features, and treatment. Heart Vessels.

[29] Peng MC, Hou CJ, Li JY, Hu PY, Chen CY (2007). Prevalence of symptomatic large pleural effusions first diagnosed more than 30 days after coronary artery bypass graft surgery. Respirology.

[30] Xu R, Barg FK, Emmett EA, Wiebe DJ, Hwang WT (2018). Association between mesothelioma and non-occupational asbestos exposure: systematic review and meta-analysis. Environ Health.

[31] Olsen NJ, Franklin PJ, Reid A, de Klerk NH, Threlfall TJ, Shilkin K, Musk B (2011). Increasing incidence of malignant mesothelioma after exposure to asbestos during home maintenance and renovation. Med J Aust.

[32] *BTS statement on malignant mesothelioma in the UK, 2007*. Thorax, 2007. 62(Suppl 2): p. ii1–ii19. 10.1136/thx.2007.087619 PMC2094726 17965072

