# Supplementary material for: Does previous asbestos exposure increase the risk of a post coronary artery bypass graft (CABG) pleural effusion – a routine data study?
Source: BMC Pulm Med. 2023 Aug 21;23:307. doi: 10.1186/s12890-023-02555-9 (PMC10441712; doi:10.1186/s12890-023-02555-9)
Supplement: Supplementary file 1 — Supplementary Material 1 [file 12890_2023_2555_MOESM1_ESM.docx]

Supplementary Tables

| **OPCS Procedure codes that indicate pleural effusion:** | |  |
| --- | --- | --- |
| T083 | Fenestration of pleura | 0 |
| T121 | Drainage of lesion of pleura NEC | 21 |
| T122 | Drainage of pleural cavity NEC | 209 |
| T123 | Aspiration of pleural cavity | 353 |
| T124 | Insertion of tube drain into pleural cavity | 552 |
| T128 | Other specified puncture of pleura | 26 |
| T129 | Unspecified puncture of pleura | 0 |

Supplementary Table 1: Numbers of pleural procedure types identified in HES dataset

|  | | | **OR (95% CI)** | **p-value** |
| --- | --- | --- | --- | --- |
| **Outcome 1:** Pleural effusion diagnosis or procedure in 30 days to 1 year after CABG; n (%) | Total | 2009 (4%) |  | |
|  | Crude model |  | 1.72 (1.32, 2.24) | <0.001 |
|  | Adjusted model |  | 1.35 (1.03, 1.77) | 0.03 |
| **Outcome 2:** Pleural effusion procedure in 30 days to 1 year after CABG; n (%) | Total | 812 (1%) |  | |
|  | Crude model |  | 2.12 (1.47, 3.08) | <0.001 |
|  | Adjusted model |  | 1.65 (1.14, 2.40) | 0.01 |

Supplementary Table 2 - Association between asbestos exposure and post-CABG pleural effusion (sensitivity analysis)

*CABG – Coronary Artery Bypass Graft; OR – odds ratio; 95% CI – 95% confidence interval*
